# Diagnostic and Therapeutic Challenges in the Management of Intermediate and Frail Elderly Multiple Myeloma Patients

**DOI:** 10.3390/cancers12113106

**Published:** 2020-10-24

**Authors:** Francesca Bonello, Mario Boccadoro, Alessandra Larocca

**Affiliations:** Myeloma Unit, Division of Hematology, University of Torino, Azienda Ospedaliero-Universitaria Città della Salute e della Scienza di Torino, 10126 Torino, Italy; francesca.bonello@edu.unito.it (F.B.); mario.boccadoro@unito.it (M.B.)

**Keywords:** multiple myeloma, frailty score, frailty-tailored treatment

## Abstract

**Simple Summary:**

Choosing the optimal therapy for elderly patients with multiple myeloma (MM) poses a difficult challenge for clinicians. Older patients are an extremely heterogeneous population, they are underrepresented in clinical trials, and data on octogenarians have been mainly limited to real-life settings. Treatment options for intermediate and frail patients might include dose-adapted combinations and less toxic combinations based on novel agents. Moreover, the discriminative power of the International Myeloma Working Group (IMWG) frailty score in detecting frailty and vulnerability could be improved by combining together aging-related factors (performance status, comorbidities, functional status) with disease-related factors (International Staging System stage, cytogenetic risk). Objective parameters could improve the reproducibility of this score and limit the subjectivity determined by patient-reported questionnaires on functional evaluations. Efforts are ongoing to simplify the IMWG frailty score and expand its use in real-life clinical practice.

**Abstract:**

Multiple myeloma (MM) mostly affects elderly patients, which represent a highly heterogeneous population. Indeed, comorbidities, frailty status and functional reserve may vary considerably among patients with similar chronological age. For this reason, the choice of treatment goals and intensity is particularly challenging in elderly patients, and it requires a multidimensional evaluation of the patients and the disease. In recent years, different tools to detect patient frailty have been developed, and the International Myeloma Working Group frailty score currently represents the gold standard. It identifies intermediate-fit and frail patients requiring gentler treatment approaches compared to fit patients, aiming to preserve quality of life and prevent toxicities. This subset of patients is underrepresented in clinical trials, and studies exploring frailty-adapted approaches are scarce, making the choice of therapy extremely challenging. Treatment options for intermediate-fit and frail patients might include dose-adapted combinations, doublets, and less toxic combinations based on novel agents. This review analyzes the available tools for the assessment of frailty and possible strategies to improve the discriminative power of the scores and expand their use in real-life and clinical trial settings. Moreover, it addresses the main therapeutic challenges in the management of intermediate-fit and frail MM patients at diagnosis and at relapse.

## 1. Introduction

Multiple myeloma (MM) is mainly a disease of the elderly, with a median age at diagnosis of 70 years. Approximately 65–70% of cases are diagnosed in people older than 65 years of age and 35–40% in people older than 75 years [1,2]. Over the past two decades, the introduction of novel agents has resulted in a substantial survival improvement of MM patients, and the recent advent of immunotherapy has further increased survival rates. Although this benefit appears to be more consistent in younger patients, it has also been observed in patients aged ≥65 years, who have been traditionally considered ineligible for high-dose therapy and transplantation. In MM patients aged 65/75 years, the 5-year overall survival (OS) improved from approximately 30% up to 50–55% until 2010–2012, whereas in the most recent data a 4-year OS of 75% was observed in patients diagnosed in the years 2013–2017 [3,4,5].

On the other hand, outcomes remain suboptimal in patients aged ≥75 years, as compared to younger ones, due to the higher incidence of toxicity resulting in morbidity, mortality and inadequate treatment exposure [6,7]. Even with novel regimens and treatment optimization (e.g., administration of subcutaneous or weekly bortezomib), discontinuation due to adverse events (AEs) occurs in about 10–15% of patients, and approximately 10% of patients aged 80 years and older experience toxic deaths [8,9,10,11].

Given these premises, choosing the optimal MM therapy for elderly patients poses a difficult challenge for clinicians. Older patients are underrepresented in clinical trials, with patients aged ≥75 years accounting for 30–40% of patients enrolled in first-line trials and, sharply, for 10–15% of patients enrolled in trials at relapse. [8,9,12,13,14,15,16,17,18]. Therefore, most of the available data have been obtained from small subgroup analyses, whereas data on octogenarians have been mainly limited to real-life settings [19]. Moreover, elderly patients represent an extremely heterogeneous population. Older age at diagnosis negatively affects survival, particularly in patients aged ≥80 years [20]. However, chronological age in itself is not enough to characterize elderly patients, since there is large variability within the same age group, reflecting differences in health and functional status. Finding reliable parameters to stratify patients according to their fitness is essential for choosing the optimal treatment and limiting toxicity. In recent years, several scores have been proposed to meet these aims. These scores help clinicians distinguish fit patients able to tolerate more intense treatment combinations (triplets, quadruplets, or even autologous transplantation) from intermediate-fit and frail patients who benefit from gentler approaches [21].

This review provides an overview of available strategies to assess patient frailty and of treatment management options for intermediate-fit and frail elderly patients.

## 2. Frailty: Definition and Assessment in MM Patients

### 2.1. Definition of Frailty

Aging is a dynamic process associated with the concomitant occurrence of multiple diseases, risk of physical and cognitive decline and increased vulnerability. Frailty is a state of increased vulnerability and inability to adapt to a stressor event that triggers disproportionate changes in health status and may result in a significant decline of functional ability [22]. The prevalence of frailty increases with age, ranging from about 10% in patients aged 75–79 years up to 26% in patients aged >85 years [23]. Fried and colleagues developed a phenotype model for the detection of frailty that considers unintentional weight loss, self-reported exhaustion, low energy expenditure, slow gait speed, and weak grip strength, thus predicting the risk of adverse outcome and mortality. The 7-year mortality rate was 45% in frail and 12% in non-frail patients [24]. Another model considered the cumulative presence of baseline individual clinical and laboratory variables to define a Frailty Index (FI), which was predictive of OS and risk of hospitalization [25,26].

The Comprehensive Geriatric Assessment (CGA) is a multidimensional evidence-based process used to detect frailty and to guide treatment decisions in older oncology patients (conventionally ≥70 years old). CGA explores social status, comorbidities, functional status, mental state, polypharmacy, nutritional status, and the presence of geriatric syndromes. This evidence may detect deficits that are not routinely assessed by clinical examination [27]. CGA was a predictor of severe treatment-related toxicities and OS in several cancers, including hematologic malignancies [28]. Nevertheless, geriatric assessment is not routinely performed by hematologists because it is time-consuming and requires a complex evaluation of multiple domains. In recent years, efforts to find reliable and easily available tools for the assessment of frailty in elderly MM patients led to the development of several scores, which are analyzed below.

### 2.2. Standardized Tools for Frailty Assessment in MM Patients

The first score for the assessment of frailty in MM patients was introduced by the International Myeloma Working Group (IMWG) in 2015 and was based on the analysis of more than 800 transplant-ineligible newly diagnosed multiple myeloma (NDMM) patients enrolled in clinical trials and treated with regimens containing lenalidomide, bortezomib, or carfilzomib. A baseline geriatric assessment was performed and consisted of three tools: the Charlson Comorbidity Index (CCI) to estimate the number and severity of comorbidities, the Katz Index of Independence in Activities of Daily Living (ADL), and the Lawton Instrumental Activities of Daily Living (IADL) scale. The self-reported ADL and IADL questionnaires were adopted to assess functional status and independence. According to an additive scoring system (0–5 points) including geriatric evaluation and age, three groups of patients were identified: “fit” (score = 0), “intermediate fitness” (score = 1) and “frail” (score ≥ 2). The IMWG frailty score was found to be predictive of progression-free survival (PFS), OS, risk of grade (G) 3–4 non-hematologic AEs, and treatment discontinuation. In detail, 3-year OS was 84% in fit patients, 76% in intermediate-fit patients (HR 1.61), and 57% in frail patients (HR 3.57). The risks of G3-4 non-hematologic AEs (HR 1.57) and treatment discontinuation were particularly higher in frail patients (HR 2.21) than in fit patients [29]. The prognostic impact of the frailty profile on OS was independent of the International Staging System (ISS) stage [30], chromosomal abnormalities and type of treatment. The score was validated in a real-life population of 125 NDMM patients treated in a single center in Germany. Again, 3-year OS was 91% for fit, 77% for intermediate-fit (HR 1.77) and 47% for frail patients (HR 5.80) [31].

Recently, the European Myeloma Network reviewed the data about frailty assessment in MM, aiming to advance this knowledge both in clinical trials and clinical practice [32]. The IMWG frailty score currently represents the gold standard to define frailty in MM patients [32]. Nevertheless, in the past years, other scores have been proposed and tested to further improve the assessment of frailty.

Kleber et al. developed the Revised Myeloma Comorbidity Index (R-MCI), implementing a previous score (the Freiburg Comorbidity Index) proposed in 2011 that considered renal and lung impairment and Karnofsky Performance Status (KPS) [33]. The R-MCI score was developed on 800 NDMM patients and included age, frailty, and cytogenetics. Combining and weighing these factors, patients with a R-MCI score ≤ 3 were defined as fit, those with a R-MCI score = 4–6 as intermediate fit, and those with a R-MCI score > 6 as frail. Compared to fit patients, intermediate-fit and frail patients showed significantly inferior PFS (median, 4.1 vs. 1.9 vs. 0.9 years) and OS (median, 10.1 vs. 4.4 vs. 1.2 years, respectively). The R-MCI score was internally validated, and similar outcomes were obtained in the validation cohort [34]. Differently from the IMWG frailty score, which included only patients evaluated as transplant-ineligible, the R-MCI was developed on a younger population (median age 63 vs. 74 years old) and included also patients receiving autologous stem-cell transplantation (ASCT, 48% of patients). The strength of the R-MCI score was the implementation of the cytogenetic risk in the model. Indeed, adverse cytogenetics was demonstrated to be an independent risk factor for survival, and it could be included in the global evaluation of MM patients together with performance status and organ function.

In 2016, the Mayo Clinic developed a score based on age, performance status, and circulating N-terminal pro-B-type natriuretic peptide (NTproBNP) levels. Around 350 NDMM patients from clinical practice were included in the analysis. Age ≥ 70 years, Eastern Cooperative Oncology Group (ECOG) performance status ≥ 2, and NTproBNP levels ≥ 300 ng/L were found to be independent predictors of survival and were included in an additive score. Four stages were identified (according to the presence of 0-1-2-3 risk factors). Median OS was not reached, 4.9, 2.3 and 1.5 years in stages I, II, III, and IV, respectively (*p* < 0.001) [35]. As in the R-MCI score population, patients were relatively young (median age 65 years), and 38% of them received ASCT. This score was relevant because of the role of NTproBNP levels as a simple, easily available and objective parameter for the detection of senescence. To our knowledge, the Mayo Clinic score has not yet been validated and no direct comparison with the IMWG frailty score has been made.

### 2.3. Emerging Tools to Improve the Frailty Assessment of MM Patients

**Figure 1:**
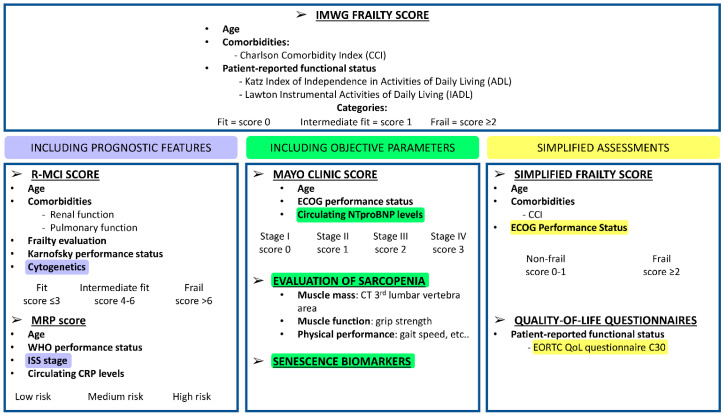
Tools for geriatric assessments in multiple myeloma patients. International Myeloma Working Group (IMWG) frailty score [29]; Revised Myeloma Comorbidity Index (R-MCI) [33]; UK Myeloma Research Alliance Risk Profile (MRP) [36]; Mayo Clinic Score [35]; evaluation of sarcopenia [37]; simplified frailty score [38,39]; quality-of-life questionnaires: European Organization for Research and Treatment of Cancer Quality of Life (EORTC QoL) questionnaire C30 [40,41]. *Abbreviations*. WHO, World Health Organization; ISS, International Staging System for Multiple Myeloma [30]; CRP, C-reactive protein; ECOG, Eastern Cooperative Oncology Group; NTproBNP, circulating N-terminal pro-B-type natriuretic peptide; CT, computed tomography; CCI, Charlson Comorbidity Index.

Despite being the gold standard for frailty assessment, the IMWG frailty score has the limitations of being time-consuming and of using subjective parameters—such as the ADL and IADL scales—that are patient-reported. Recently, efforts have been made to improve the discriminative power of the IMWG frailty score and to simplify the detection of frailty and biological vulnerability in MM patients (Figure 1).

In 2019, data from NDMM patients enrolled in the randomized Myeloma IX and Myeloma XI trials (ISRCTN.com Registry identifiers: ISRCTN68454111 and ISRCTN49407852) were used to design the UK Myeloma Research Alliance Risk Profile (MRP) [36 The prognostic variables of the score were the World Health Organization (WHO) performance status, age, ISS stage, and circulating levels of C-reactive protein (CRP). The MRP score identified patients at low risk, medium risk and high risk for OS (median 60 vs. 40 vs. 25 months, respectively) and early mortality (OR 2.14 for medium risk and OR 4.76 for high risk vs. low risk). This score remained predictive regardless of the type of treatment administered (alkylating agents, immunomodulatory drugs and proteasome inhibitors) and cytogenetic risk [36]. The strengths of this score are the incorporation of objective variables (i.e., CRP, ISS stage) and its simplicity, since it does not require any supplemental investigations in addition to parameters routinely assessed in clinical practice. The risk profile defined by this score included both tumor characteristics (ISS stage) and host characteristics (performance status, age, CRP). The MRP score was retrospectively validated in real-life patients and will be prospectively validated and compared with the IMWG Frailty Score in the ongoing UK-MRA Myeloma XIV study (FiTNEss) [42].

A simplified frailty score was developed by Facon and colleagues [38] on patients enrolled in the FIRST trial comparing lenalidomide-dexamethasone (Rd) vs. melphalan-prednisone-thalidomide (MPT). This score considered age and comorbidities according to the CCI and ECOG performance status, and it divided patients into frail and non-frail groups. This score was found to be predictive of PFS (HR 1.36 for frail vs. non-frail groups), OS (HR 1.86), treatment discontinuation (HR 1.66), as well as G3-4 hematologic (HR 1.16) and non-hematologic AEs (HR 1.18) [38]. The rationale for this simplified score was based on the use of performance status as a surrogate for the evaluation of the patient’s functional reserve, which in the IMWG frailty score was assessed through the ADL and IADL questionnaires [39]. However, performance status is a quantitative functional assessment tool, rather than a qualitative tool, and does not reflect the heterogeneity of comorbidities and functional abilities of patients.

Another possibility to simplify the IMWG frailty score was explored by the HOVON group, which evaluated physical functioning using the first five questions of the European Organization for Research and Treatment of Cancer Quality of Life (EORTC QoL) questionnaire C30 [40,41]. This tool was less time consuming and complex than the ADL and IADL questionnaires and demonstrated to identify frail patients. However, it was evaluated in a small patient population, and further confirmation is needed.

An objective indicator of frailty that is gaining interest in the geriatric evaluation of MM patients is sarcopenia. The term “sarcopenia” describes a condition of progressive and generalized decline in muscle mass and function that is associated with an increased likelihood of adverse outcomes [41]. The prevalence of sarcopenia among people aged 65 years or older is 5–10%, reaching 38% in cancer patients [43,44]. Muscle mass is assessed by imaging techniques, the gold standard of which is computed tomography of the area of the 3rd lumbar vertebra. Muscle function is assessed by grip strength and physical performance, the latter being measured by different tests, one of the most standardized of which is considered to be the gait speed test. Sarcopenia was evaluated in elderly MM patients enrolled in the HOVON 123 trial, and results showed that loss of muscle mass was more frequent in frail vs. intermediate-fit patients (22% vs. 5%). Low muscle mass, but not muscle function, was associated with impaired clinical outcome (HR for treatment discontinuation 2.84; HR for OS 2.76) [37]. Further evaluation of sarcopenia in elderly MM patients is being carried out in the HOVON 143 trial.

Finally, laboratory-based biomarkers of frailty represent an area of interest, despite being still at a very experimental stage in MM. Markers of cellular senescence (including markers of DNA damage, telomere length, and cell cycle arrest) and inflammation associated with aging are being explored [32]. For instance, the capacity of DNA repair decreases with frailty and parameters of increased DNA breakage (such as increased phosphorylated H2AX levels) could be integrated as objective tools for frailty detection [45]. Another study demonstrated significantly reduced telomere length in frail patients and its association with an increased risk of death [46]. Preliminary studies are also investigating the role of expression levels of the marker of cellular senescence p16^INK4a^, as well as biomarkers of sarcopenia such as myostatin and insulin-like growth factor levels [32,47,48].

## 3. Treatment Considerations for Intermediate-Fit and Frail Patients at Diagnosis

The assessment of frailty can be used to guide treatment decisions in terms of type, intensity, and goals of therapy. In fit patients, treatment is aimed at achieving deep and durable responses (complete response or minimal residual disease negativity) and includes full-dose triplets/quadruplets and even ASCT in eligible patients. On the contrary, the prevention of toxicity and the preservation of quality of life are essential for intermediate-fit and frail patients [21,49]. Outcomes of main phase III trials in elderly MM patients at diagnosis are summarized in Table 1.

The addition of daratumumab to standard treatments such as Rd and bortezomib-melphalan-prednisone (VMP) radically changed the treatment scenario for transplant-ineligible patients [8,9,56]. A longer PFS, also in patients older than 75 years of age, was observed both in the daratumumab-Rd arm vs. the Rd arm (HR 0.63) in the MAIA trial and in the daratumumab-VMP arm vs. the VMP arm in the ALCYONE trial (HR 0.53). However, no data about the frailty status of enrolled patients were available. The addition of daratumumab resulted in a higher rate of G ≥ 3 AEs in the overall population, particularly in terms of infections (23% vs. 15% in the ALCYONE trial and 32% vs. 23% in the MAIA trial). In the MAIA trial, neutropenia (50% vs. 35%) and fatigue (8% vs. 4%) were also observed. Whether these full-dose combinations may be feasible for all elderly patients regardless of frailty status remains to be clarified, and more data are needed.

Some evidence suggested that frail patients might benefit more from two-drug regimens than from full-dose triplets. Results of the EMN01 trial showed no differences in terms of PFS and OS in intermediate-fit and frail patients receiving Rd vs. the triplet melphalan-prednisone-lenalidomide (MPR) and cyclophosphamide-prednisone-lenalidomide (CPR). On the contrary, fit patients obtained a better outcome with MPR (HR for PFS of MPR vs. Rd 0.72, *p* = 0.04) [53,54]. The phase III UPFRONT trial compared the doublet bortezomib-dexamethasone (Vd) vs. VMP vs. bortezomib-thalidomide-dexamethasone (VTD) in real-life, transplant-ineligible patients (42% of whom were aged ≥75 years). No differences in terms of PFS and OS were observed among the three arms (median PFS, 14.7, 15.4, and 17.3 months respectively, *p* = 0.46; median OS, 49.8, 51.5, and 53.1 months respectively, *p* = 0.79) [51]. Vd was better tolerated, with fewer G ≥ 3 hematologic toxicities compared to VMP (thrombocytopenia 2% vs. 15%) and fewer drug discontinuations compared to VTD (29% vs. 38%).

Another option is to reduce treatment intensity over time, thus sparing toxicity and allowing patients to remain in treatment longer. This approach was evaluated in the RV-MM-PI-0752 trial, which compared continuous Rd treatment with nine Rd cycles followed by lenalidomide maintenance at a lower dose (10 mg) and discontinuation of dexamethasone in intermediate-fit patients. Data presented so far showed comparable PFS (PFS rate at 20 months, 42% vs. 43%) and OS (OS rate at 20 months, 85% vs. 79%) between the experimental arm and the standard arm. On the other hand, event-free survival accounting for both efficacy (disease progression or death) and safety (G4 hematologic AEs, G3-4 non-hematologic AEs and lenalidomide discontinuation) was significantly longer in the experimental arm than in the standard arm (median 9.3 vs. 6.6 months, *p* = 0.04) [57]. This type of dose- and schedule-adjusted approach may deserve further evaluation in intermediate-fit and frail patients. The ongoing IFM_2017-03 trial is evaluating a similar steroid-sparing strategy by comparing the doublet daratumumab-lenalidomide to Rd (ClinicalTrials.gov Identifier: NCT03993912). The combination of bortezomib, lenalidomide and dexamethasone (VRD) demonstrated to be superior to Rd alone, albeit more toxic [55]. A dose-reduced VRD regimen (VRD-lite) for nine cycles followed by VR consolidation discontinuing dexamethasone was evaluated in elderly patients (median age, 73 years old). Median PFS was 35.1 months and, despite the limitations of cross-trial comparisons, it was similar to the median PFS observed in patients ≥75 years old treated with standard VRD in the SWOG S0777 trial. Although 78% of patients had to reduce at least one drug, only 4% of patients discontinued treatment due to toxicity [58]. Preliminary results of the HOVON 123 trial showed that dose-adjusted VMP administered to frail patients for six cycles instead of nine cycles induced comparable response rates and was feasible in 70% of patients. On the contrary, only 54% of frail patients in the control arm completed nine cycles. In this light, a shorter induction followed by low-dose maintenance can be a feasible strategy, since prolonged treatment might expose frail patients to excessive toxicity.

Finally, another option could be the use of “non-frail drugs” (e.g., drugs that are included in standard regimens for fit patients) in less toxic drug combinations. The triplet daratumumab, ixazomib and low-dose dexamethasone is currently under evaluation in a phase I/II trial on intermediate-fit and frail patients. Preliminary data showed an overall response rate (ORR) of 74% and 78% in intermediate and frail patients and a median PFS of 23 and 12 months, respectively. Preliminary analysis showed that discontinuation rates and toxic deaths were low in intermediate-fit patients, but reached 7% and 9% in frail patients, suggesting that this latter group might benefit from even gentler approaches [59].

Ongoing trials will further evaluate the frailty-adjusted approach. The FITNESS trial (ClinicalTrials.gov Identifier: NCT03720041) will compare a non-frailty-adjusted induction treatment with ixazomib-lenalidomide-dexamethasone (IRd) to dose-adjusted induction treatment with IRd according to frailty status.

## 4. Treatment Considerations for Intermediate-Fit and Frail Patients at Relapse

Although many treatment options are available at relapse nowadays, there is no consensus on the optimal treatment strategy for intermediate-fit and frail patients beyond the first line of therapy. The first remission is the most important phase, since not all patients receive a second line, due to physical/functional conditions or residual toxicities from the previous line. Indeed, real-world data reported that approximately 61% of patients received 2 or more lines of therapy during their disease course [60]. Poor performance status (ECOG > 1, OR 1.38, 95% CI 1.20–1.59) and older age (>65 years old, OR 1.22, 95% CI 1.06–1.39) negatively impacted the probability of receiving a second line [61]. An analysis of octogenarians from real-life clinical practice reported that only 33% of patients received a second line of treatment, mainly bortezomib-based (43%) [19].

In clinical trials on relapsed/refractory (RR)MM, patients aged 75 years or older represent a minority of study populations (10–15%) [15,16,17,18,62]. Since patients with significant comorbidities are usually not eligible for enrollment, study populations do not generally reflect the characteristics of real-world populations. Moreover, data on frailty assessment have not yet been collected, and all of the available scores have been developed on NDMM patients and never evaluated at relapse. Taken together, these elements make evidence-based treatment recommendations for intermediate-fit and frail patients at relapse extremely challenging. The following evidence comes from small subgroup analyses of elderly patients (conventionally aged ≥75 years) enrolled in clinical trials. The advantage in PFS and OS observed with triplets and new-generation agents in comparison with old standard treatments is maintained in elderly patients, although higher toxicity is usually observed in older vs. younger subjects (Table 2).

In the ASPIRE trial, PFS in patients aged ≥75 years was significantly longer in the carfilzomib-lenalidomide-dexamethasone (KRD) arm vs. the Rd arm (median, 30.3 vs. 16.6 months, HR 0.62) [17,63]. Older patients receiving KRD experienced greater cardiovascular toxicity, as compared to younger patients (G ≥ 3 AEs 14% vs. 5%) [64]. Similarly, in the ENDEAVOR trial, median PFS in patients aged ≥75 years was 19 vs. 9 months (HR 0.38) in the carfilzomib-dexamethasone (Kd) arm vs. the bortezomib-dexamethasone (Vd) arm, and translated into an OS advantage as well (36.1 vs. 23.9 months, respectively, HR 0.78). Nevertheless, G ≥ 3 cardiovascular AEs in the Kd arm were more frequent in older than in younger patients (10.4% vs. 3.6%) [18,65]. These data suggest a careful selection of elderly patients before receiving carfilzomib-based treatments.

The triplet IRd is an appealing option for intermediate-fit and frail patients, given its fully oral administration. The phase III TOURMALINE-MM1 trial compared IRd vs. Rd, reporting a PFS advantage with the triplet also for patients aged ≥75 years (median, 18.5 vs. 13.1 months, HR 0.87), with good safety profile and quality of life in the overall population [62].

The addition of daratumumab to Rd (POLLUX trial) and Vd (CASTOR trial) more than doubled the PFS compared to both Rd and Vd in patients aged ≥75 years (median PFS of daratumumab-Rd vs. Rd, 28.9 vs. 11.4 months, HR 0.27; median PFS of daratumumab-Vd vs. Vd, 17.9 vs. 8.1 months, HR 0.26) [15,16,66,67,68]. Toxicity was similar to that observed in younger patients. G3-4 infusion-related reactions (IRRs) were slightly higher in ≥75-year-old patients compared to younger patients in the POLLUX trial (13.8% vs. 4.9%), but not in the CASTOR trial (10% vs. 8.5%) [67].

Finally, in the ELOQUENT-2 trial, the addition of the monoclonal antibody elotuzumab to Rd resulted in a PFS advantage over Rd. In patients older than 75 years, the advantage in terms of PFS and OS was maintained, and elotuzumab did not increase the toxicity of Rd, showing that this triplet is a valid option for non-fit patients [69,73].

Beyond the second line of therapy, data about the treatment of elderly patients are even more scarce. In the phase III ICARIA-MM trial, heavily pretreated patients were randomized to receive the anti-CD38 monoclonal antibody isatuximab with pomalidomide and dexamethasone (Isa-Pd) vs. Pd. Median PFS was significantly longer with Isa-Pd and was similar in patients aged ≥75 years (11.4 vs. 4.5 months, HR 0.49), 65–74 years (11.6 vs. 8.6 months, HR 0.64), and <65 years (11.5 vs. 5 months, HR 0.66). Patients aged ≥75 years experienced higher rates of G3–4 AEs and treatment discontinuation compared to younger patients. Interestingly, IRRs were rarer in patients aged ≥75 years than in those aged 65–74 years and <65 years (28% vs. 36% vs. 42%, respectively) [70]. Data about other pomalidomide-based combinations (such as elotuzumab-Pd vs. Pd and bortezomib-Pd vs. Pd) in ≥75-year-old patients are scarce. Nevertheless, compared to Pd, these triplets proved to reduce the risk of progression or death also in this subgroup of patients, although results did not reach statistical significance [71,72]. The addition of low-dose cyclophosphamide to Pd could be another option, given the better efficacy in comparison with Pd alone (median PFS 4.4 vs. 9.5 months), without a significant increase in toxicity and the advantage of being a fully oral combination [74]. Nevertheless, data about this combination are limited to phase I/II studies.

These data suggest that triplet combinations including novel, less toxic agents may also be used in intermediate-fit and selected frail patients, although a careful patient selection is needed. On the other hand, in frail patients with poor clinical conditions, low-dose doublets or a palliative approach with low-dose thalidomide, cyclophosphamide or melphalan ± corticosteroids could be used for symptomatic relief [21,75].

## 5. Conclusions

The evaluation of elderly patients with MM requires to stage not only the disease, but also patients’ aging. The IMWG frailty score allowed clinicians to delve into the complexity of frailty in MM. Nevertheless, efforts are needed to further optimize this tool. The discriminative power of the score in detecting frailty and vulnerability could be improved by combining together aging-related factors (performance status, comorbidities, functional status) with disease-related factors (ISS stage, cytogenetic risk). Objective parameters could improve the reproducibility of this score and limit the subjectivity determined by patient-reported questionnaires on functional evaluations. In this direction, the evaluation of sarcopenia represents the most promising tool, whereas laboratory biomarkers (such as p16INK4a, myostatin and Insulin-like Growth Factor 1) are still at a very experimental stage [76]. Efforts are ongoing to simplify the IMWG frailty score, in order to expand its use in real-life clinical practice. To this end, it would be possible to substitute the ADL and IADL scales, which are time-consuming, with easier and more rapid questionnaires. The substitution of patient-reported questionnaires with a performance status has also been considered. However, whether ADL/IADL scales could be replaced by a performance status is highly debated, since performance status is not considered discriminative enough, and the advantage of physician-derived over patient-derived scales is controversial. In the IMWG frailty score, the performance status did not affect OS, whereas the frailty status substantially increased the risk of death, thereby suggesting that a more sophisticated evaluation procedure of elderly patients could be necessary [29]. Another open issue is the role of chronological age in scoring systems. Recently presented data showed that patients who were determined to be frail by age only (e.g., >80 years old) had outcomes similar to those of patients who were determined to be frail due to comorbidities and functional impairments (median OS 42.9 vs. 41.6 months, *p* = 0.54). This suggests that, beyond a certain age, patient vulnerability is independent of other factors [77]. Given these premises, new/improved scores should be evaluated prospectively, validated and possibly compared to the IMWG frailty score, also considering real-life populations.

Despite the growing emphasis on frailty assessment, to date data on frailty-tailored treatment remain scarce. We still need to gather evidence about the optimal management of a large population of elderly patients not described in clinical trials. Real-life data suggest that only half of the octogenarians diagnosed with MM have received first-line therapy, although this percentage has been increasing in the last years. Nevertheless, among octogenarian MM patients receiving treatment, the benefit was similar to that observed in younger patients (70–79 years old). The median OS of octogenarian MM patients receiving treatment vs. those referred to supportive care is 21.4 months vs. 6 months [78]. When looking at goals of therapy in these delicate patients, we need to consider tolerability and, consequently, quality of life during treatment as key factors. Preservation of independence can be more important than prolonged survival if the latter comes with long-term toxicities and the need for frequent and prolonged hospital visits. Data on elderly patients receiving chemotherapy showed that about 60% of them considered their current quality of life to be more important than long-term survival, and up to 80% of them considered their current mental/functional state to be more important than long-term survival as well [79]. The inclusion of quality-of-life measures in clinical trials is recent, but is progressively gaining more interest in this field.

In conclusion, the optimal management of non-fit patients is a difficult challenge for hematologists. Ongoing and future trials on transplant-ineligible NDMM patients should include frailty assessments, quality-of-life measures, and personalized treatment approaches according to patient frailty. Moreover, frailty assessment measures should be evaluated in the relapse setting, possibly considering residual toxicities from previous treatments.

## Figures and Tables

**Table 1:** Treatment outcome in ≥75-year-old patients enrolled in phase III clinical trials at diagnosis.

TRIAL	Regimen	Age, Median (Range); Pts Aged ≥75 Years, %	Median PFS in the Overall Population, Months	Median PFS in Pts Aged ≥75 Years, Months	Main G ≥ 3 Adverse Events * in the Overall Population, %	Discontinuation Due to Adverse Events in the Overall Population, %
VISTA [12,50]	VMP vs. MP	71 (48–91) 30%	24 vs. 16.6 m HR 0.48 (95% CI nr)	na HR 0.53 (95% CI 0.31–0.90)	Neutropenia 40% vs. 38%; thrombocytopenia 38% vs. 31%; neuropathy 13% vs. 0	15% vs. 14%
UPFRONT [51]	Vd vs. VTd vs. VMP	73 (66–79) 42%	14.7 vs. 15.4 vs. 17.3 m *p* = 0.46	na	Neutropenia 2% vs. 3% vs. 19%; thrombocytopenia 2% vs. 4% vs. 15%; infections 21% vs. 16% vs. 18%; neuropathy 22% vs. 27% vs. 20%	29% vs. 38% vs. 34%
FIRST [7,13,52]	Rd vs. MPT	73 (40–92) 35%	25.5 vs. 21.2 m HR 0.72 (95% CI 0.61–0.85)	20.3 vs. 19.8 m HR 0.80 (95% CI 0.62–1.03)	Neutropenia 30% vs. 45% thrombocytopenia 9% vs. 11% infection 32% vs. 17%	12% vs. 14%
EMN01 [53,54]	Rd vs. (MPR/CPR)	73 (50–91) 35%	21 vs. 22 m HR 1.10 (95% CI 0.9–1.35)	na * intermediate fit: 20 vs. 22 m * frail: 22 vs. 18 m	Neutropenia 25% vs. 64% vs. 29%; thrombocytopenia 7% vs. 18% vs. 9%; infection 9% vs. 11% vs. 6.5%	14% vs. 18% vs. 15%; * in intermediate-fit pts: 18% vs. 20% vs. 13%; * in frail pts: 18% vs. 23% vs. 30%
SWOG S0777 [55]	VRd vs. Rd	63 (56–71) 31% NTE	43 vs. 30 m HR 0.71 (95% CI 0.56–0.91)	39 vs. 20 m, *p* = ns HR na	Hematologic 49% vs. 49%; infections 18% vs. 13.7%; neurologic 34.6% vs. 11.3%	na
ALCYONE [8]	Dara-VMP vs. VMP	71 (40–93) 30%	36.4 vs. 19.3 m HR 0.42 (95% CI 0.34–0.51)	nr vs. 20.4 m HR 0.53 (95% CI 0.32–0.85)	Neutropenia 39.9% vs. 38.7%; thrombocytopenia 34.4% vs. 37.6%; infections 23.1% vs. 14.7%; neuropathy 1.4% vs. 4%; infusion reaction 4.9% vs. na	4.9% vs. 9%
MAIA [9]	Dara-Rd vs. Rd	73 (45–90) 43%	nr vs. 31.9 m HR 0.56 (95% CI 0.43–0.73)	nr vs. 31.9 m HR 0.63 (0.44–0.92)	Neutropenia 50% vs. 35.3%; infection 32.1% vs. 23.3%; infusion reaction 2.7% vs. na	7.1 vs. 15.9%
CLARION [14]	KRd vs. VMP	72 (42–91) 31%	22.3 vs. 22.1 m HR 0.9 (95% CI 0.75–1.01)	na HR 0.81 (95% CI 0.58–1.12)	Neutropenia 22.6% vs. 29.4%; thrombocytopenia 15.4% vs. 21.1%; respiratory infection 12.6% vs. 11.7%; hypertension 9.1% vs. 3%; cardiac failure 5.1% vs. 1.5%; renal failure 7.2% vs. 2.1%; neuropathy 0.2% vs. 9.4%	16.7% vs. 14.7%

Abbreviations: Pts, patients; PFS, progression-free survival; m, months; HR, hazard ratio; CI, confidence interval; *p*, *p*-value; na, not available; nr, not reached; ns, not significant; VMP, bortezomib, melphalan, prednisone; MP, melphalan, prednisone; Vd, bortezomib, dexamethasone; VTd, bortezomib, thalidomide, dexamethasone; Rd, lenalidomide, dexamethasone; MPT, melphalan, thalidomide, prednisone; MPR, melphalan, prednisone, lenalidomide, CPR, cyclophosphamide, prednisone, lenalidomide; VRd, bortezomib, lenalidomide, dexamethasone; NTE, transplant-ineligible; Dara, daratumumab; KRD, carfilzomib, lenalidomide, dexamethasone; G, grade. * Including adverse events of clinical interest for the specific drug administered.

**Table 2:** Treatment outcome in ≥75-year-old patients enrolled in phase III clinical trials at relapse.

TRIAL	Regimen	Age, Median (Range); Pts aged ≥ 75 Years, %	Median PFS in the Overall Population, Months	Median PFS in Pts Aged ≥ 75 Years, Months	Main G ≥ 3 Adverse Events *in Pts Aged ≥75 Years	Discontinuation Due to Adverse Events in Pts Aged ≥75 Years
ASPIRE [17,63,64]	KRd vs. Rd	64 (31–91) 12%	26.3 vs. 17.6 m HR 0.69 (95% CI 0.57–0.83)	30.3 vs. 16.6 m HR 0.62 (95% CI 0.36–1.08)	[in pts aged ≥70 years]: neutropenia 36.9% vs. 23.2%; thrombocytopenia 20.45 vs. 25.2%; pneumonia 15.5% vs. 14.3%; hypertension 5.8% vs. 1.8%; cardiac failure 8.7% vs. 1.8%	[in pts aged ≥70 years]: 34% vs. 34.8%
ENDEAVOR [18,65]	Kd vs. Vd	65 (30–89) 15%	18.7 vs. 9.4 m HR 0.53 (95% CI 0.44–0.65)	18.7 vs. 8.9 m HR 0.38 (95% CI 0.22–0.64)	Thrombocytopenia 7.8% vs. 9.2%; cardiac failure 10.4% vs. 3.1%; hypertension 11.7% vs. 3.1%; pneumonia 7.8% vs. 12.3%	26% vs. 35.4%
TOURMALINE-MM1 [62]	IRd vs. Rd	66 (30–91) 15%	20.6 vs. 14.7 m HR 0.74 (95% CI 0.59–0.94)	18.5 vs. 13.1 m HR 0.87 (95% CI na)	na	na
POLLUX [66,67]	Dara-Rd vs. Rd	65 (34–89) 11%	44.5 vs. 17.5 m HR 0.44 (95% CI 0.35–0.55)	28.9 vs. 11.4 m HR 0.27 (95% CI 0.10–0.69)	Neutropenia 44.8% vs. 31.4%; thrombocytopenia 10.3% vs. 14%; pneumonia 17.2% vs. 11.4%; pulmonary embolism 3.4% vs. 11%; infusion reaction 13.8%	17% vs. 17%
CASTOR [67,68]	Dara-Vd vs. Vd	64 (30–88) 12%	16.7 vs. 7.1 m HR 0.31 (95% CI 0.24–0.39)	17.9 vs. 8.1 m HR 0.26 (95% CI 0.10–0.65)	Thrombocytopenia 45% vs. 37.1%; neuropathy 10% vs. 5.7%; diarrhea 10% vs. 0; pneumonia 15% vs. 17%; infusion reaction 10%	17% vs. 20%
ELOQUENT-2 [69]	Elo-Rd vs. Rd	66 (37–91) 20%	19.4 vs. 14.9 m HR 0.44 (0.59–0.86)	Na HR 0.63 (95% CI 0.41–0.96)	na	na
ICARIA-MM [70]	Isa-Pd vs. Pd	67 (36–86) 20%	11.5 vs. 6.5 m HR 0.60 (95% CI 0.44–0.81)	11.4 vs. 4.5 m HR 0.48 (95% CI 0.24–0.95)	neutropenia 50% vs. 46.4%; thrombocytopenia 15.6% vs. 10.7%; infection 46.9% vs. 35.7%; infusion reaction 3.1%	15.6% vs. 14.3%
ELOQUENT-3 [71]	Elo-Pd vs. Pd	67 (36–81) 21%	10.3 vs. 4.7 m HR 0.54 (95% CI 0.34–0.86)	Na HR 0.62 (95% CI 0.23–1.67)	na	na
OPTIMISMM [72]	PVd vs. Vd	68 (59–73) 16%	11.2 vs. 7.1 m HR 0.61 (95% CI 0.49–0.77)	Na HR 0.78 (0.46–1.32)	na	na

Abbreviations: Pts, patients; PFS, progression-free survival; m, months; HR, hazard ratio; CI, confidence interval; *p*, *p*-value; na, not available; RD, carfilzomib, lenalidomide, dexamethasone; Rd lenalidomide, dexamethasone; Kd, carfilzomib, dexamethasone; Vd, bortezomib, dexamethasone; IRd, ixazomib, lenalidomide, dexamethasone; Dara, daratumumab; Elo, elotuzumab; Isa, isatuximab; Pd, pomalidomide, dexamethasone; PVd, pomalidomide, bortezomib, dexamethasone; G, grade.
